# Evidence of dipstick superiority over urine microscopy analysis for detection of hematuria

**DOI:** 10.1186/s13104-016-2240-y

**Published:** 2016-09-08

**Authors:** Aurélien Bataille, Morgane Wetzstein, Alexandre Hertig, Sophie Vimont, Eric Rondeau, Pierre Galichon

**Affiliations:** 1 Inserm UMR_S 1155, Hôpital Tenon, Paris, France; 2 Urgences Néphrologiques et Transplantation Rénale, Hôpital Tenon, 4, rue de la Chine, 75020 Paris, France; 3 Service de Bactériologie, Hôpital Tenon, Paris, France; 4 Université Pierre et Marie Curie (Paris 6), Sorbonne Universités, Paris, France

**Keywords:** Screening test, Hematuria, Sensitivity

## Abstract

**Background:**

There is an unresolved debate on the best screening method for hematuria as a symptom of glomerulonephritis or urological malignancies. The urinary dipstick is generally considered as an imperfect surrogate for urine microscopy analysis.

**Results:**

We designed a study to compare urine microscopy analysis, urinary dipstick and flow cytometry, using controlled dilutions of blood in urine samples from volunteers collected in two different physiologically-relevant conditions (basal state and hyperhydration). We found that although all techniques were 100 % effective in detecting hematuria at basal state, these results were variably reproduced when testing the same final amount of hematuria in urine collected after hyperhydration. Our data shows a variable sensitivity for the detection of hematuria by urine microscopy analysis or flow cytometry, but not by urinary dipstick.

**Conclusions:**

Urinary dipstick qualifies as a better screening test for hematuria than urine microscopy analysis or flow cytometry, as it is sensitive and performs better in unstandardized conditions. It is universally available and also faster and cheaper than cytometric techniques.

**Electronic supplementary material:**

The online version of this article (doi:10.1186/s13104-016-2240-y) contains supplementary material, which is available to authorized users.

## Findings

### Background

Screening for hematuria is recommended in many situations, including screening of high risk patients in occupational medicine [1–3] and tests are expected to be sensitive. Missed or delayed diagnosis of hematuria may lead to neglecting urological (cancer) or nephrological (glomerulonephritis) life-threatening diseases [4]. There has long been a debate as to whether urine dipstick or urine microscopy analysis (UMA) is the preferred testing method, but there is no consensus amongst practitioners [5, 6]. UMA performance may be altered by red blood cell (RBC) degradation in urine, and urine dipstick may misdiagnose hemoglobinuria or myoglobinuria as hematuria [6]. To our knowledge, no controlled study has compared urine dipstick and UMA to an independent gold standard, and studies evaluating the urine dipstick using UMA as a benchmark do not evaluate the diagnostic performance of UMA itself. Our objective was to compare urine dipstick and UMA as a diagnostic test for hematuria, using urine samples with a known concentration of RBC.

### Methods

We obtained a calibration by diluting a controlled amount of blood in urine. Control urine was collected from volunteers (healthy young males to avoid genital blood contamination) after informed oral consent. 50 mL midstream urine was collected at baseline and after 1.5 L of water ingestion. Blood was taken from one volunteer, diluted in urine to 1:10^3^, and ten fold serial dilutions in urine were performed up to 1:10^8^. Urine dipstick (Multistix® 8SG, Siemens) with an automatic analyser (Clinitek Status®) yielded semi-quantitative results (0, prints, +, ++, +++, Additional file 1). Urine microscopic analysis at a 400-fold optical magnification (Leitz Wetzlar® Ortholux) in 1 µL counting chambers allowing quantitative per volume assessment of hematuria [7] and flow cytometry (MACSQuant® Analyser, Miltenyi Biotec) gating RBC on a side scatter versus forward scatter plot yielded a count/mm^3^ (Additional files 2 and 3 respectively). Data are summarized as median and interquartile range. Two-sided Wilcoxon tests were performed at 0.05 significance level, and paired when appropriate using R statistical package (version 2.14.1).

**Fig. 1:**
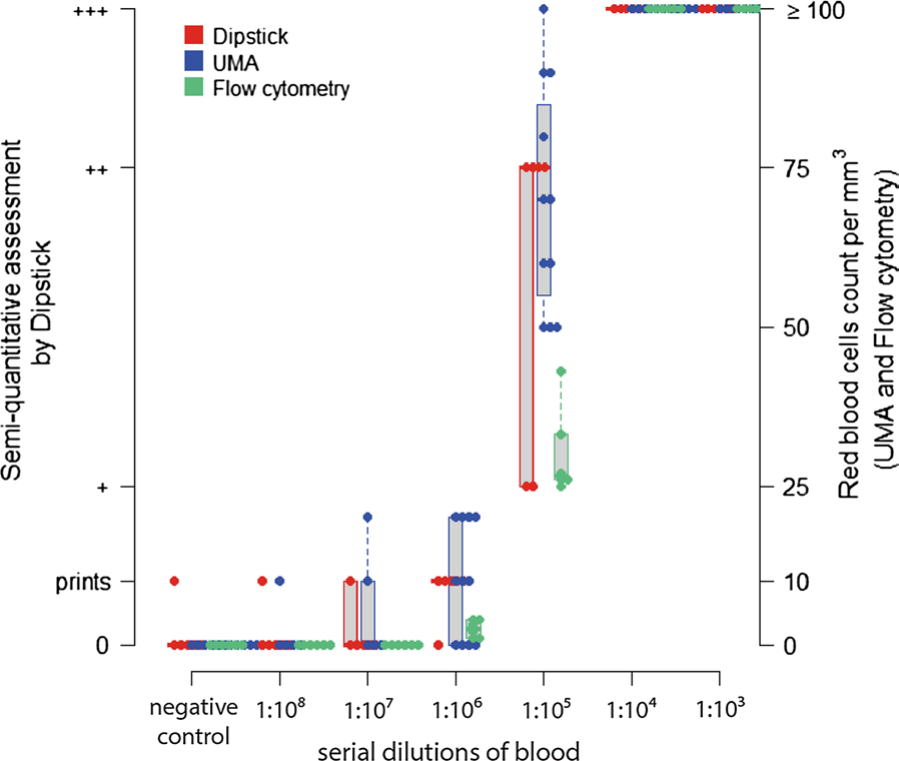
Detection of hematuria by urine dipstick, UMA or flow cytometry at increasing concentrations of RBC

Using serial dilutions, we found that urine dipstick, UMA and flow cytometry detected hematuria at the threshold of 1:10^6^ blood dilution, with the greatest sensitivity for flow cytometry (100 %), followed by urine dipstick (83 %) and UMA (64 %). At the 1:10^5^ dilution, all three techniques had 100 % sensitivity (Fig. 1), and we thus decided to use this dilution to compare the robustness of the three different test depending on hydration.

### Results

**Fig. 2:**
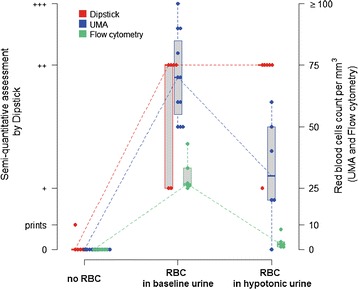
Results of UMA, urine dipstick and flow cytometry for hematuria in baseline and hypotonic urine

We collected urine after water ingestion to evaluate the three methods in a different clinical setting and to compare to baseline control urine. As expected, we found that urine density decreased after water load [1010 (1006; 1010) vs. 1030 (1022; 1030), p = 0.034]. When we assessed the three different methods in baseline and hypotonic urine, using the same 1:10^5^ dilution, we found that the cytomorphological tests (UMA and flow cytometry) were not reproducible, with a systematic underestimation of hematuria (57 % median fold decrease, p = 0.036 and 92 % median fold decrease, p = 0.031, respectively) whereas assessment by the urine dipstick conserved its sensitivity (++ positivity) (Fig. 2).

We compared the sensitivity of three different methods for diagnosing hematuria, and the robustness of these methods when there is a clinically relevant change in urine composition. Urine dipstick was more sensitive than UMA, especially in hypotonic urine. The enhanced sensitivity of the dipstick is explained by its ability to detect heme even after RBC lysis. We performed the blood dilutions with urine collected at basal state and after hydration to ensure that the chemical and physical conditions were relevant to patients with hematuria. This is very relevant to urological hematuria (i.e. a bleeding occurring directly in urine), and perhaps less to nephrological hematuria (where the RBC journey with primary urine in the tubules). We chose to evaluate the detection of hematuria independently of the effect of changes in diuresis, but this parameter could enhance the effect of hydration that we observed: in patients, hypotonic urine is often associated with increased diuresis and urine dilution could further decrease the sensitivity of hematuria detection. The dipstick can be performed immediately, but UMA needs to be brought to the laboratory first, and delay also reduces the sensitivity of UMA [8].

### Conclusions

Thus, instead of considering the urine dipstick as a surrogate marker for UMA, it should be the preferred method for screening hematuria. Timed urine samples were once considered to be the gold standard, but UMA is now recommended by most guidelines. We believe that UMA may not be reliable if performed in unstandardized conditions, and that it should rather be a confirmatory test performed in controlled conditions (midstream urine harvested in the morning when it is more acid and hypertonic), to rule out isolated pigmenturia and to detect dysmorphic RBC. The urine dipstick should, therefore, be preferred for the screening of hematuria as it is more sensitive and cheaper than UMA.

## Additional files

10.1186/s13104-016-2240-y BU2.csv contains the dipstick data. "nom" stand for individual numbers, "H" for basal morning urine, "h" for urine after hydration, "densité" for urine density, and "10 px" for the 10^x^ red blood cell concentrations.
10.1186/s13104-016-2240-y ECBU2.csv contains the microscopy analysis data. "nom" stand for individual numbers, "H" for basal morning urine, "h" for urine after hydration, "densité" for urine density, and "10 px" for the 10^x^ red blood cell concentrations.
10.1186/s13104-016-2240-y FACS2.csv contains the flow cytometry data. "nom" stand for individual numbers, "H" for basal morning urine, "h" for urine after hydration, "densité" for urine density, and "10 px" for the 10^x^ red blood cell concentrations.

## Abbreviations

UMA: urine microscopy analysis
RBC: red blood cells
